# “How did it go?”—investigation of physician’s experience during embryo transfer and subsequent pregnancy outcomes

**DOI:** 10.1007/s10815-025-03609-z

**Published:** 2025-08-02

**Authors:** Sara C. Pierpoint, Mary Peavey, Linnea Goodman

**Affiliations:** 1 Dept. of Obstetrics and Gynecology, University of Virginia Medical Center, 1215 Lee Street, Charlottesville, VA 22903 USA; 2 Atlantic Fertility, 10208 Cerny Street, Suite 306, Raleigh, NC 27617 USA; 3 Virginia Fertility and IVF, 4100 Olympia Cir Ste 201, Charlottesville, VA 22911 USA

**Keywords:** Embryo transfer, Fertility treatment, In vitro fertilization, Patient counseling, Patient-centered care, Surgeon perception, Ultrasound guidance

## Abstract

**Purpose:**

This research sought to investigate if a physician’s experience of an embryo transfer (ET) correlates to rates of success.

**Methods:**

A single experienced physician rated characteristics of ETs including ease of transfer (1–10), visibility (1–10), presence of blood, and amount of mucus (0–3) at the time of ET. Main outcomes were positive beta-human chorionic gonadotropin level 11 days post transfer and ongoing pregnancy rate.

**Results:**

A total of 365 transfers were rated. The average age of patients was 34.2 + / − 4.6 years. A total of 69.6% of cycles were programmed, and 73.2% of the transferred embryos underwent pre-implantation genetic testing for aneuploidy (PGT-a) prior to transfer. Average ease of transfer scores (9.3 + / − 1.7 vs 9.2 + / − 1.7; *p* = 0.71) and visibility scores (8.3 + / − 2.3 vs 8.1 + / − 2.8; *p* = 0.37) were similar between positive and negative pregnancy tests, respectively. Blood and mucus did not influence pregnancy rates. There was no correlation between ease of mock and actual embryo transfers. Multivariate logistic regression for positive bHCG demonstrated a significant predictive value for PGT-a (OR 2.50, 95% CI 1.47–4.23). Ease of transfer (OR 1.02, 95% CI 0.88–1.15), visibility (OR 1.04, 95% CI 0.94–1.15), mucus (OR 1.00, 95% CI 0.77–1.30), and embryo age (OR 0.98, 95% CI 0.92–1.03) were not predictive.

**Conclusions:**

In experienced hands, physician subjective impressions of transfer characteristics were not significantly correlated with a successful transfer.

## Introduction

An embryo transfer (ET) is a clinically unique procedure, involving the precise placement of a 0.1–0.2 mm microscopic embryo with the patient, provider, sonographer, and embryologist observing in real time [1]. Ongoing innovations, including improved embryo selection, optimized endometrial preparation, and increasingly atraumatic catheter designs, continue to refine success rates [2].

Clinicians performing ETs often develop a subjective impression of each transfer based on ease of catheter passage, visualization under ultrasound, presence of blood or mucus, and other procedural factors. However, whether these real-time perceptions have predictive value for implantation and pregnancy outcomes remains unclear. Physicians frequently reflect on whether an “easy” or “clean” transfer is more likely to succeed, but current literature offers limited guidance on the prognostic utility of these impressions [3–6].

Several studies have attempted to correlate technical difficulty with pregnancy rates [7–9], and a 2023 meta-analysis of 15 studies involving more than 29,000 transfers supported these findings, reporting that difficult transfers were associated with significantly reduced clinical pregnancy rates [10]. However, the analysis was limited by variation in provider experience, lack of standardized definitions, and inconsistent documentation of transfer quality.

The use of ultrasound guidance during ET is well established as a means of improving outcomes and has become standard practice in many fertility centers [10, 11]. Later investigations assessed whether the method of image acquisition, by patient or sonographer, impacted success but found no significant difference [12].

Notably, most prior studies have isolated specific aspects of the ET process or included multiple providers with diverse skill levels and techniques, making it difficult to assess the overall significance of a physician’s composite impression of the transfer. The current study seeks to address this gap by prospectively evaluating whether a single experienced physician’s real-time ratings of ET characteristics, including ease, visibility, and presence of blood or mucus, correlate with pregnancy outcomes. Establishing whether physician perception carries predictive value may help guide post-transfer counseling and contribute to a more nuanced understanding of ET success factors.

## Materials and methods

At an academically affiliated private practice, IRB approval was obtained, and a single experienced physician prospectively recorded subjective characteristics of embryo transfers between January 2023 and September 2024. The provider scored each transfer on four characteristics: subjective ease of transfer on a scale of 1 difficult–10 easy, visibility by transvaginal ultrasound (1 no visibility–10 perfect), presence or absence of blood on the catheter, and amount of cervical mucus (0 none–3 copious). Characteristics were selected based on existing literature and for what the physician and laboratory suspected could be contributing factors. A 1–10 scale was selected for ease of transfer and visualization for an intuitive classification. Mucus was characterized on a smaller scale due to less variability, and blood was a dichotomous characterization due to previous literature commenting merely on its presence. Additional information collected at the time of transfer included patient demographics, pre-implantation genetic testing for aneuploidy (PGT-a) status, embryo and patient age, and type of cycle stimulation (programmed or natural).

A single resident physician reviewer not involved with the transfers retrospectively collected data on trial embryo transfer that was performed on all patients within 6 months of actual embryo transfer. Three attending providers performed these trial transfers. The reviewer classified each trial transfer as “easy,” “moderate,” or “difficult” based on the procedure note. This was correlated to the actual transfer, with ease of transfer also being segregated into easy (score 8–10), moderate (score 4–7), or difficult (score 1–3).

Programmed cycles were performed using leuprolide acetate and/or oral contraceptive pill suppression followed by oral estrogen administration with subsequent intramuscular progesterone in oil and vaginal progesterone. Natural cycles were either completely unmedicated or were augmented with oral ovulation induction agents. Trigger with beta-human chorionic gonadotropin was administered when a lead follicle was greater than or equal to 17 mm. Progesterone was then initiated, and embryo transfer was performed 6 or 7 days following the trigger based on LH values. Embryo transfers were performed in an operating room adjacent to the embryology laboratory. Bedside transabdominal ultrasound was performed by nursing staff. After insertion of the speculum, a soft Q-Tip was used to swipe excess mucus from the cervical os if present. A flushing catheter was then inserted into the lower cervix and flushed with 1–2 ccs of embryo media. A mock transfer was performed using a Wallace embryo transfer catheter to the internal cervical os. The actual transfer was then performed using the afterload technique with the embryo placed approximately 1 cm from the uterine fundus.

Primary outcome was positive bHCG level 11 days post transfer. Secondary outcome was ongoing pregnancy at 8 weeks gestation. Statistics were performed with the student’s *t*-test or chi-squared as appropriate, with *p* < 0.05 considered significant. Multivariate logistic regression was performed, including difficulty, visibility, mucus, embryo age, and PGT-a.

## Results

There was a total of 365 transfers, with an average patient age of 34.2 +/− 4.6 years and embryo age of 33.2 +/− 4.5 years. Demographics are outlined in Table 1. The most common diagnosis among the cohort was unexplained infertility (26.3%), followed by male factor (23.3%), ovulatory and oocyte dysfunction (14.3%), social (10.1%), multifactorial (7.7%), tubal factor (6.6%), endometriosis (4.1%), uterine factor (2.7%), genetic (2.5%), and recurrent pregnancy loss (2.5%).

**Table 1:** Comparison of average scores for subjective characteristics and PGTa between those that resulted in a positive vs. negative pregnancy test. Means +/− SD

Variable	Total (*n* = 365)	+ bHCG (*n* = 273)	- bHCG (*n* = 92)	*P*-value
Age (years)	34.2 ± 4.6	34.2 ± 4.6	34.2 ± 4.4	0.97
Embryo age (years)	33.2 ± 4.5	33.2 ± 4.5	32.2 ± 4.5	0.98
BMI (kg/m^2)	26.5 ± 6.1	26.7 ± 6.2	25.8 ± 2.0	0.25
Endometrial lining (mm)	9.8 ± 1.8	9.9 ± 1.7	9.5 ± 2.0	0.05
Mock scored as moderate or difficult (*n*)	62 (16.9%)	43 (15.8%)	19 (20.7%)	0.94
Transfer scored as moderate or difficult (*n*)	30 (8.2%)	22 (8.1%)	8 (8.7%)	0.71
Ease of transfer score	9.3 ± 1.7	9.3 ± 1.6	9.2 ± 1.7	0.71
Visibility score	8.3 ± 2.4	8.3 ± 2.3	8.1 ± 2.8	0.37
+ Blood (*n*)	16 (4.4%)	14 (5.1%)	2 (2.2%)	0.23
High mucus (*n*)	62 (17.0%)	48 (17.6%)	14 (15.2%)	0.78

The vast majority of transfers were frozen blastocysts (97.0%) and of a single embryo (94.0%) and were genetically euploid by PGT-a testing (73.2%). Most of the transfers (69.6%) were programmed, with the remainder natural. A total of 273 patients (74.5%) had an initial positive bHCG value 11 days after transfer, and 248 patients (67.9%) had an ongoing pregnancy at 8 weeks gestation. There were 20 chemical pregnancies (5.2%) and five spontaneous abortions (1.4%).

**Fig. 1:**
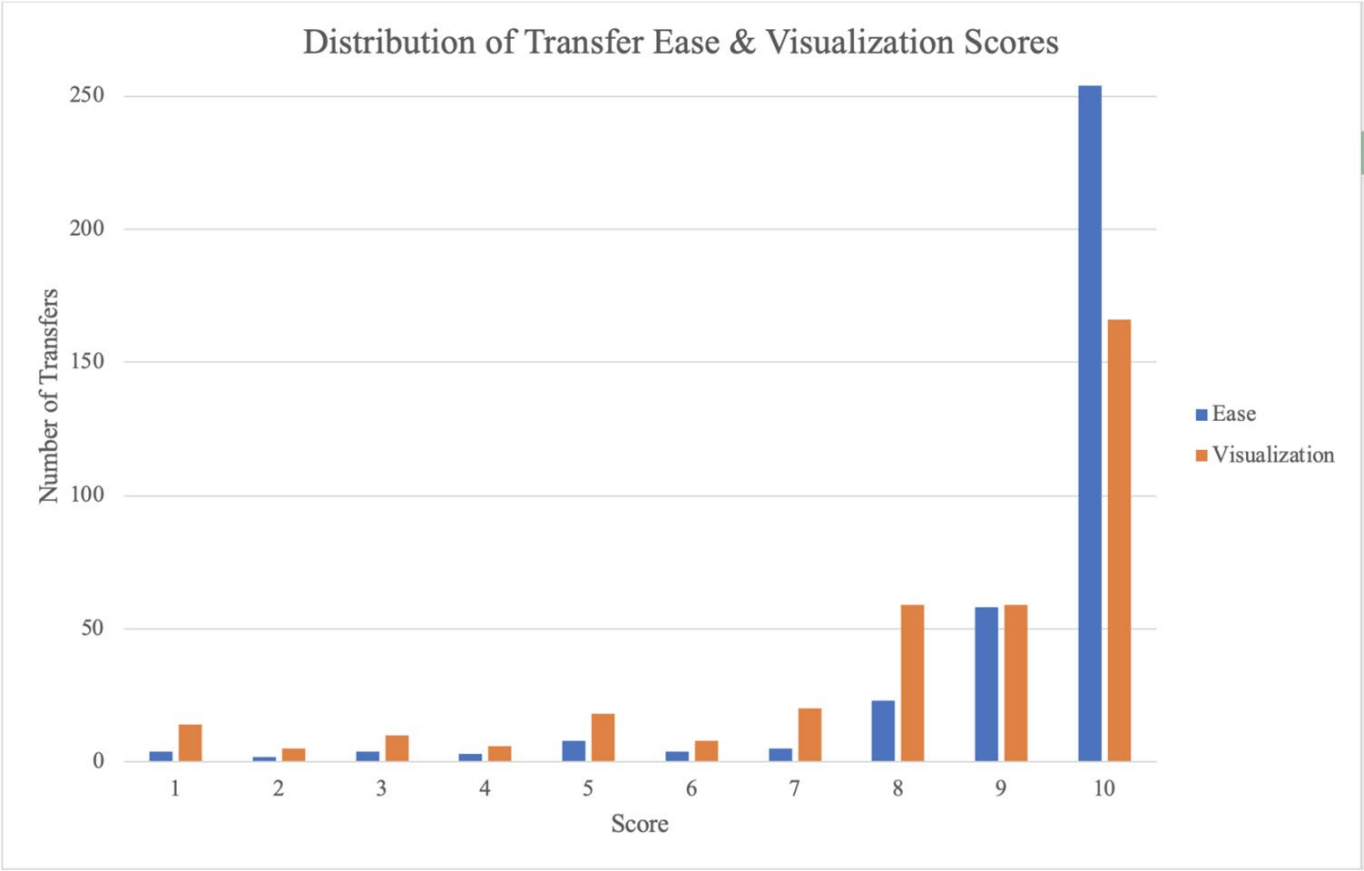
Distribution of ease of transfer and visualization scores for all the embryo transfers. Scores were assigned (1-most difficult to 10-easiest)

Regarding subjective characteristics, most transfers were considered both easy to perform and easy to visualize. Only 10 transfers (2.7%) were considered difficult (score 1–3), and 20 (5.5%) were considered moderate (score 4–7). There were 53 (14.5%) transfers that had a suboptimal visualization score (less than or equal to 5). The distribution of transfer ease and visibility scores is depicted in Fig. 1. The average difficulty scores between transfers that resulted in positive and negative pregnancy tests were similar (9.3 +/− 1.7 vs 9.2 +/− 1.7; *p* = 0.71). Among the 10 transfers with a difficult rating, 70% had a positive pregnancy test. When expanded to include both moderate and difficult, 22 of the 30 (73.3%) transfers resulted in a positive pregnancy test. Two patients who had difficult transfers required a stylet, and both subsequently had positive test results.

**Fig. 2:**
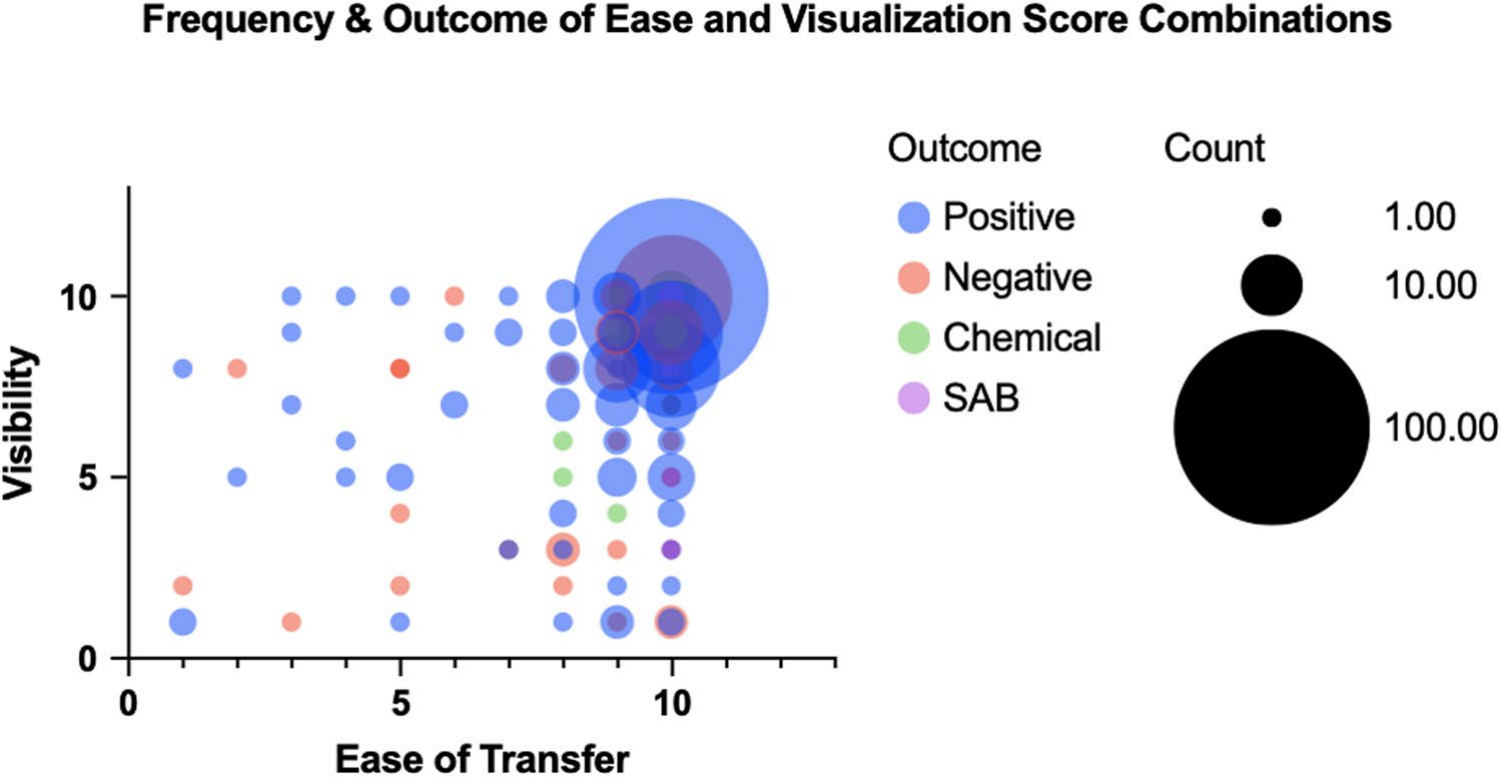
Frequency of ease and visibility score combinations along with pregnancy outcome

The visibility scores were similar between transfers that resulted in positive and negative pregnancy tests (8.3 +/− 2.3 vs 8.1 +/− 2.8; *p* = 0.37). Of the 53 transfers with suboptimal visualization, the pregnancy rate was 69.8%, which was similar to those with optimal visualization. Visibility was positively correlated with ease of transfer (*r* = 0.39, *p* < 0.01) and negatively with BMI (*r* =  − 0.27, *p* < 0.01). Figure 2 demonstrates frequency of ease and visibility score combinations along with pregnancy outcome. The largest cohort, 100 patients (27.4%), scored 10 in both transfer ease and visibility and had a positive outcome.

Neither the presence of blood on the catheter (only present in 4.4% of transfers) nor a high amount of cervical mucus correlated with the difference between outcomes, as listed in Table 1. A majority of the transfers were the patient’s first, with the rest being subsequent transfers, and there was no significant difference in outcome between primary and subsequent transfers (*p* = 0.17).

Multivariate logistic regression for positive bHCG demonstrated a significant predictive value for PGT-a (OR 2.50, 95% CI 1.47–4.23). Ease of transfer (OR 1.02, 95% CI 0.88–1.15), visibility (OR 1.04, 95% CI 0.94–1.15), mucus (OR 1.00, 95% CI 0.77–1.30), and embryo age (OR 0.98, 95% CI 0.92 − 1.03) were not predictive.

When evaluating the trial embryo transfer to the actual embryo transfer, the majority of both mock and real transfers were considered easy, 82.5% and 91.5%, respectively. Significantly more mock transfers were considered moderate difficulty (14.0% vs 4.7%, *p* < 0.01), but similar numbers of difficult mock and real transfers were observed (3.3% vs 3.6, *p* = 0.84). There was no difference in ease of transfer score for mock transfer between those with and without a positive bHCG (*p* = 0.60). Additionally, there was not a correlation between mock and real embryo transfer score (*r* = 0.19, *p* < 0.01).

## Comment

### Principal finding

This study found that physician impressions of embryo transfer, rated by difficulty, visibility, presence of blood, and amount of mucus, were not predictive of pregnancy outcomes. None of these variables were significantly associated with a positive β-hCG level at 11 days or with ongoing pregnancy at 8 weeks. The only factor significantly associated with a positive outcome was the use of PGT-a.

## Results in the context of what is known

The relationship between transfer difficulty and outcome has been examined for decades, though definitions of “difficult” have varied considerably [9]. Many studies classify difficulty based on the need for adjunctive instruments such as a tenaculum or stylet [13–15]. A 2019 observational study categorized difficulty by resistance encountered during catheter passage and found no significant correlation with clinical outcomes [13]. This finding aligns with a 2013 study that reported higher pregnancy rates with easy versus difficult transfers (38.1% vs 21.4%, *p* < 0.05) [14]. However, a 2021 study by Inal et al. found that catheter type, used as a proxy for difficulty, did not impact implantation or pregnancy rates [16].

Other investigators have defined transfer difficulty based on patient anatomy. Larue et al. (2017, 2020) identified challenging features such as cervical tortuosity, endocervical crypts, polyps, and uterine positioning as predictors of difficulty. Despite these anatomic classifications, their subsequent outcomes study showed no significant difference in clinical pregnancy rates between easy and difficult groups [17, 18].

Subjective provider assessments have also been used. Studies by Listigono et al. (2013) and Plowden et al. (2016), which evaluated 6000 and 1000 transfers, respectively, found that provider-rated difficult transfers had significantly lower clinical pregnancy (24.6% vs 30.7%; 35.7% vs 48.7%) and live birth rates (19.5% vs 25.3%; 26.6% vs 41.2%) [19].

In contrast, our study found no association between subjective markers of transfer quality and pregnancy outcomes. Consistent with previous findings, most transfers were rated as easy [19]. Importantly, our study shows that even when a transfer was perceived as more difficult, outcomes remained comparable, offering reassurance to both providers and patients in such cases.

The presence of blood during ET has also been variably associated with poor outcomes. A 2003 study of over 600 cycles reported decreased implantation and pregnancy rates when blood was seen on the catheter [20], a finding echoed by a 2012 retrospective study noting an 11.8% reduction in clinical pregnancy with blood present (*p* = 0.03) [21]. However, other studies have not confirmed this association [22, 23]. Our findings align with the latter group, suggesting that blood does not necessarily impair success.

Of all characteristics evaluated, the lack of correlation between visibility and pregnancy outcome is particularly noteworthy. Ultrasound guidance is standard for ET, but our data suggest that image quality does not impact success. This has clinical relevance in patients with challenging anatomy or elevated BMI, where optimal visualization may be difficult. While increased BMI was associated with lower visibility scores, it was not associated with reduced pregnancy rates. These findings can reduce anxiety for both providers and patients when image quality is suboptimal.

Finally, there was no correlation between the ease of mock transfers and actual transfers. This may indicate that when a mock transfer is challenging, the physician adapts accordingly to improve the actual transfer.

## Clinical implications

Embryo transfer is a high-stakes, emotionally charged moment in an IVF cycle. Patients are awake and attuned to every detail, often looking to their provider’s reaction for clues about success. These findings provide a useful counseling tool: even if a transfer is perceived as difficult, it does not predict failure. Providers can reassure patients both before and after ET that procedural impressions, positive or negative, are not determinative of outcome.

## Research implications

This study opens the door to further investigations. Expanding this research to include multiple providers would allow for intra- and inter-provider comparisons and help assess whether individual perceptions vary in predictive value. Additionally, using transfer duration as an objective measure of difficulty may offer further insight. The apparent disconnect between mock and actual transfers also merits more exploration to assess the utility of mock ETs in clinical practice.

## Strengths and limitations

The prospective, single-provider design of this study is both a strength and a limitation. By using a single experienced physician to assess embryo transfers in real time, the study minimizes variability in technique, perception, and clinical environment. This design better reflects the lived clinical experience and improves internal consistency. However, because the provider was not blinded to patient history, including prior failed cycles or known anatomical challenges, there is a possibility of subconscious bias. Notably, if such bias existed, it would be expected to increase concordance between physician perception and outcome, which was not observed.

One limitation is the absence of a priori power or sample size calculation. As a result, the study may be underpowered to detect subtle but clinically meaningful differences in outcome. This raises the possibility of a type II error (failing to detect an effect that may truly exist), which should be acknowledged in interpreting these findings.

Generalizability may also be limited. Only 4% of transfers in this cohort were rated as more than moderately difficult (score < 5), whereas prior studies report a higher incidence of difficult transfers [14]. Each provider may apply their own internal scale of difficulty, and this variability underscores the subjective nature of such assessments.

Additionally, embryo morphology and grade, known contributors to implantation potential, were not included in this analysis. While most embryos were euploid based on PGT-a, and only embryos graded as “good” or “fair” were considered suitable for transfer, the potential influence of embryo quality on outcomes could not be assessed and may be a confounding factor.

Nonetheless, this study’s design eliminates many previously published confounding variables, including differences in physician skill, instrumentation, and laboratory or procedural environments. It is the first study to examine whether a provider’s real-time perception of transfer quality independently predicts outcome and may help inform provider-patient counseling in everyday clinical practice.

## Conclusions

Embryo transfers can be challenging and emotionally charged, particularly when procedural difficulties arise. This study is the first to prospectively evaluate a physician’s subjective impression of ET quality and its association with outcomes. In experienced hands, transfer characteristics such as difficulty, visibility, or presence of blood or mucus were not predictive of success. These findings can reassure providers and patients alike that even imperfect or difficult transfers can lead to healthy pregnancies. Future studies involving multiple physicians and standardized difficulty metrics will help further validate and generalize these findings.

## Data Availability

Data with patient information and outcomes is sensitive and therefore kept confidential. Outcome data regarding embryo transfers for this site are available in the SART database.
